# Noncommunicable diseases among urban refugees and asylum-seekers in developing countries: a neglected health care need

**DOI:** 10.1186/1744-8603-10-24

**Published:** 2014-04-03

**Authors:** Ahmed Hassan Amara, Syed Mohamed Aljunid

**Affiliations:** 1 Institute of Tropical Medicine and International Health, Charité-Universitätsmedizin Berlin, Spandauer Damm 130, Haus 10, Berlin D-14050, Germany; 2 United Nations University International Institute for Global Health (UNU-IIGH), UNU-IIGH Building, UKM Medical Centre, Jalan Yaacob Latiff, Cheras 56000, Kuala Lumpur, Malaysia; 3 International Centre for Casemix and Clinical Coding (ITCC-UKKMC), Faculty of Medicine, Universiti Kebangsaan Malaysia, Jalan Yaacob Latiff, Cheras 5000, Kuala Lumpur, Malaysia

**Keywords:** Refugee, Asylum-seeker, Noncommunicable disease, Developing countries, Urban, Health care

## Abstract

With the increasing trend in refugee urbanisation, growing numbers of refugees are diagnosed with chronic noncommunicable diseases (NCDs). However, with few exceptions, the local and international communities prioritise communicable diseases. The aim of this study is to review the literature to determine the prevalence and distribution of chronic NCDs among urban refugees living in developing countries, to report refugee access to health care for NCDs and to compare the prevalence of NCDs among urban refugees with the prevalence in their home countries. Major search engines and refugee agency websites were systematically searched between June and July 2012 for articles and reports on NCD prevalence among urban refugees. Most studies were conducted in the Middle East and indicated a high prevalence of NCDs among urban refugees in this region, but in general, the prevalence varied by refugees’ region or country of origin. Hypertension, musculoskeletal disease, diabetes and chronic respiratory disease were the major diseases observed. In general, most urban refugees in developing countries have adequate access to primary health care services. Further investigations are needed to document the burden of NCDs among urban refugees and to identify their need for health care in developing countries.

## Introduction

During the last four decades, millions of people have fled their homes and sought asylum in other countries. According to the United Nations High Commissioner for Refugees (UNHCR) [1], by the end of 2010, there were about 15.4 million refugees and approximately 0.85 million asylum-seekers worldwide. Nearly 80 per cent of the refugees and asylum-seekers are located in developing countries (mostly in sub-Saharan Africa and Asia). The international community and host countries have been successful in helping refugees and asylum-seekers (hereafter collectively referred to as refugees) who have settled in camps or camp-like settings. Today, refugees move into cities and urban locations in anticipation for good living conditions and services, such as health care and education [2]. Recent data show that half of the world’s refugees live in non-camp settings [3], whereas in urban areas, the number of refugees almost doubled by the end of 2009, surpassing the number of refugees in camps [4]. Refugees are not always welcomed into urban areas of the host country, and usually live in shantytowns and slums in and around cities where they compete for services with other immigrants and the autochthonous urban poor. The change in refugee demographics has consequences for refugee policies, protection and the provision of services, including health care. UNHCR has responded to the change in refugee settlement by revising its 1997 policy on refugees in urban areas. The new policy from 2009 recognises urban locations as legitimate places for refugees to reside and emphasises the responsibility of UNHCR to provide protection and services to refugees [5].

The world is increasingly urbanising as people are moving from rural areas to cities, especially in developing countries. More than 60 per cent of the world’s population is projected to live in urban areas and more than 50 per cent of them are likely to be poor [6]. The same trend is expected for refugees in developing countries [7], where cities and towns are expanding and growing fast towards refugee camps. Moreover, refugees who flee from cities tend to seek refuge in urban areas. The opportunities to find work, education, health care and better livelihoods are greater in cities, and act as pull factors for refugees towards urban areas. As a result, refugees become part of and are affected by urbanisation. According to The United Nations Human Settlements Programme (UN-HABITAT) estimates, 5.3 million displaced people, including refugees, asylum-seekers, internally displaced persons and other forms of migrants are now living in cities in the developing world, particularly in sub-Saharan Africa and Western and Southern Asia [8]. In addition to urbanisation, ageing of refugees in some protracted and relatively stable situations, creates an epidemiological shift from infectious to chronic diseases [2,9]. A similar epidemiological transition is occurring in the general populations of developing countries [10]. Information on age distribution among urban refugees in developing countries is limited, possibly owing to a lack of enumeration and refugee mobility. However, data from UNHCR show that elderly populations (60 years and above) constitute two to four per cent of urban refugees in Africa, Asia and Latin America and are older than those living in camps [4].

Globally, NCDs are the leading cause of death. Approximately 80 per cent of deaths linked to NCDs occur in developing countries [11]. And although communicable diseases remain the main cause of death in most developing countries, the probability of death from NCDs, particularly in urban areas, is greater than that in the developed world [12,13]. The incidence of NCDs is predicted to increase more rapidly in developing countries than elsewhere in the world [14]. The common health problems of refugees are psychological disorders, injuries, infectious diseases, under-immunisation in children and under-managed chronic conditions such as hypertension, diabetes and chronic pain [15-18]. Chronic NCDs are now becoming a concern, particularly in middle-income populations that are affected by conflicts [3]. For example, in the case of Iraqi refugees, NCDs were the predominant health problems [19]. Similar health problems were reported during the Balkan crises [20,21]. In both situations, the international community faced numerous challenges to attend to refugees’ health care needs. The management of chronic health conditions is expensive and depletes the already limited resources available for refugee health care [17]. The health care strategy and policy for the UNHCR and other aid agencies are based on experiences from camp settings, where refugees are easily accessible [22].

Moreover, health care delivery to refugees in cities is not an easy task, even in developed countries [23,24]. Therefore, in the urban areas of developing countries, refugees’ access to health care and other services cannot be guaranteed owing to limited resources, the hidden and scattered nature of the population, a lack of security and cultural and language barriers. Sometimes legal and recognition aspects are also obstacles for refugees to receive health care even in countries with good health care systems (e.g., Malaysia). Typically, refugees and asylum-seekers do not have similar rights for accessing health care as the local population and some host governments do not assure the safety of refugees. Only ten per cent (compared to 85 per cent of the camp-based refugee population) of urban refugees had access to public health assistance in 2007 [19]. Refugees come from different countries and have different experiences with, understandings of and expectations for health and health care [25]. Accordingly, their health needs may require more than basic primary health care [26]. The primary health care available is usually not sufficient to address most chronic diseases, such as cardiovascular disease (CVD), diabetes and cancer, which require prolonged care and expensive treatment [27,28]. The shortcomings of international policies to address the needs of refugees in urban locations were highlighted during the experience with Iraqi refugees in Middle Eastern cities [29]. Furthermore, many chronic health problems perceived by refugees as not emergencies are ignored [30] due to preoccupation with other needs (food, shelter, employment, legal status) or are overlooked by health care providers owing to a lack of plans or capacities.

Despite the challenges to urban refugee health care delivery, some communicable diseases (e.g., HIV/AIDS and tuberculosis) receive attention from host countries in the interest of national public health. However, the provision of care for refugees suffering from chronic illnesses and requiring specialised consultations, expensive medications, health education and preventive health services is not adequate [5,29-31]. With respect to refugee health research, communicable diseases and mental health conditions have been studied in the context of refugees. However, NCDs among refugees (except mental health conditions) in developing countries are not adequately addressed. Routine medical screening of refugees arriving (30-90 days post-arrival) in resettlement countries have found different rates of NCDs [18,32,33]. The aim of this study is to review the literature to determine the prevalence and distribution of chronic NCDs among urban refugees living in developing countries and to describe refugee access to health care for NCDs. We will also compare the prevalence of NCDs among urban refugees with the prevalence in their home countries, when data are available, to evaluate the impact of refugee and asylum-seeker status on prevalence.

## Methods

**Figure 1:**
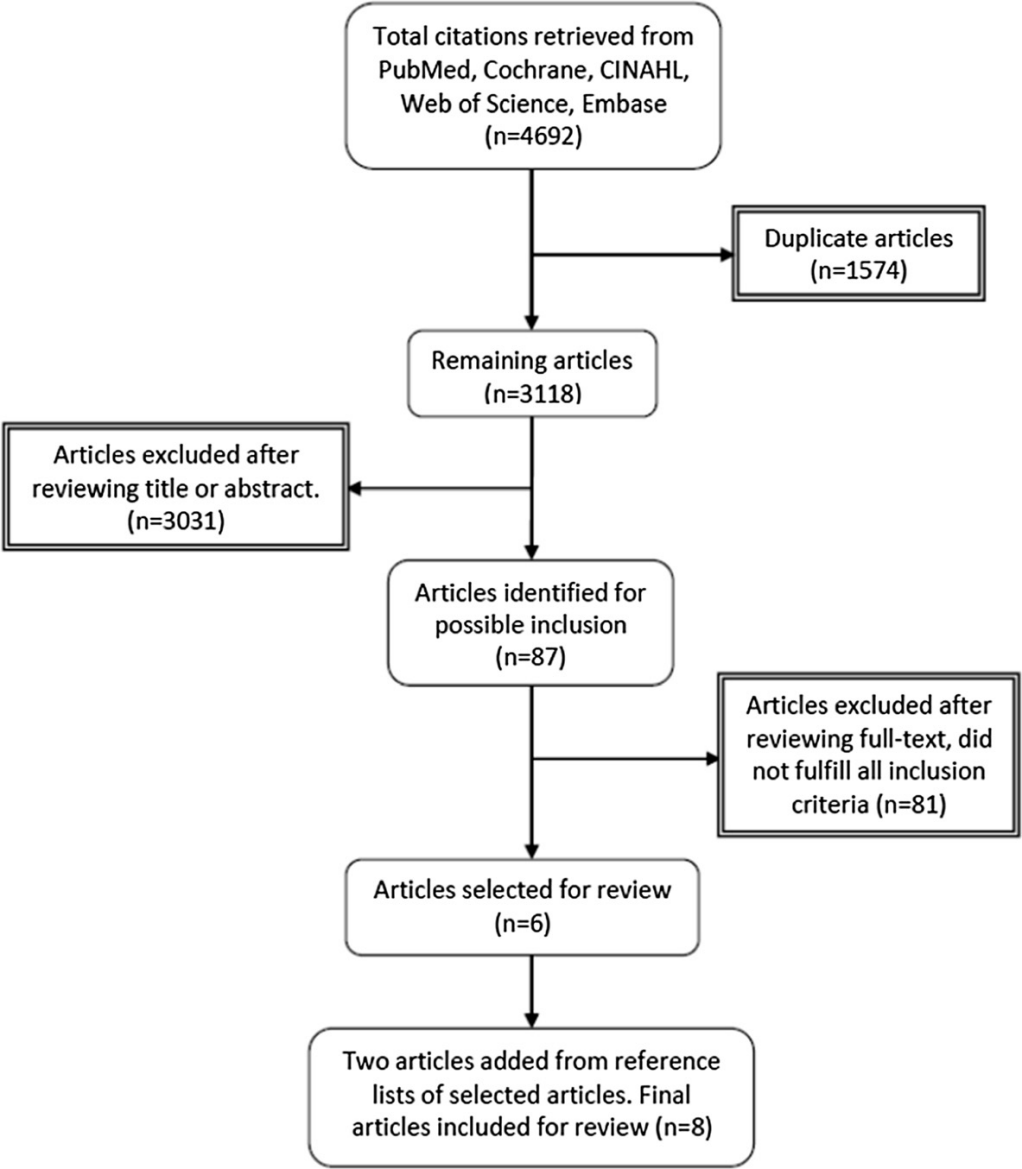
Search strategy and results.

The PubMed, CINAHL, Cochrane, Embase and the Web of Science databases were systematically searched for relevant published articles and reports on refugees and asylum-seekers from January 1980 to July 2012. The search terms included “refugee”, “asylum-seeker”, “noncommunicable disease”, “urban” and “developing countries”. Searches of similar terms, such as “refugee”, “forced migrant”, “ immigrant”, “displaced person” and “asylum-seeker”, were combined with “noncommunicable disease”, “chronic disease”, “chronic symptom”, “chronic illness”, “non-infectious disease” and “urban”, “city”, “town”, “metropolitan” and “ developing country”, “low-middle income”, “poor country”. A search was also executed for major NCDs: hypertension, diabetes mellitus, cancer and chronic pulmonary disease. Noncommunicable disease categorisations by UNHCR’s morbidity reporting were also used. If no refugee setting was mentioned, studies were included if they were conducted in countries where refugees are predominantly urban (e.g., the Middle East, Malaysia). Articles and reports on NCDs were included for review if the focus was on refugees in developing countries, the study was in an urban setting and English language was used. Conference abstracts were also included. Articles and reports that did not mention refugee setting as well as viewpoints and discussion papers were excluded. Studies were also excluded if they were primarily about refugee mental health or disabilities (physical and mental), chronic malnutrition and related conditions, or genetic and hereditary diseases. The initial search identified 87 articles for possible inclusion in the final review after duplicate articles and articles that did not fulfil the inclusion criteria were excluded (Figure 1). We excluded one study because it presented the same results of another study included in the review. Reference lists of studies that met the inclusion criteria were also scrutinised. Final articles were included after assessing the eligibility of the full-text (if available) and were reviewed based on the relevance to the review objectives. The final sample of articles included six journal articles (two from reference lists), one conference abstract and one report. Studies were either on chronic diseases among refugees or on health status and health problems with data on prevalence of NCD. The articles selected for review had studies conducted in the following countries: Jordan, Syria, Lebanon, South Korea and Turkey. Most studies were among Iraqi refugees in the Middle East. The population of other refugees and asylum-seekers were from the Palestinian Occupied Territories, North Korea, Iran, Afghanistan, Somalia and Ethiopia. A total of 44,468 refugees and asylum-seekers were involved in the studies (and two studies used the same sample to investigate two different NCDs). The following organisations’ websites were searched: UNHCR, World Health Organisation (WHO), International Organisation for Migration (IOM) and UN-HABITAT. Only the data from UNHCR met the inclusion criteria. All public health, nutrition, HIV and WASH global fact sheets for 2011 [34] with separate data on urban refugees were reviewed in addition to a few other reports from previous years. The reports were from the public health, nutrition, HIV and WASH factsheets for Asia, Central Africa, West Africa, the Middle East, North Africa, southern Africa, East Africa and the Horn of Africa and the 2008 annual report for the East and North Africa region. The UNHCR reports were on disease prevalence among approximately 1.5 million urban refugees and asylum-seekers.

## Results

Few research articles met the criteria for inclusion; 75 per cent involved Iraqi refugees. Three studies used retrospective health information data from the registers of a UNHCR partner, the UNHCR and IOM [35-37], three studies were cross-sectional population-based surveys [38-40] and one study was a case series [41]. Mateen et al. [36,42] used the same sample to investigate different NCDs. With the exception of the study from Ankara in Turkey, the data in all the studies were collected within six years (2007-2012). Table 1 presents the types of studies and refugee populations included.

**Table 1:** Studies/articles included in the review

**Author/year**	**Title**	**Year of Study/data**	**Study country**	**Origin of refugee population**	**Age group studied**	**Sample size**	**Study type**	**NCD(s) reported**	**Overall prevalence of NCD (%)**
Yaman et al. 2002 [35]	Health problems among UN refugees at a family medical centre in Ankara, Turkey	1997-1998	Turkey (Ankara)	(Iraq, Iran, Palestine Afghanistan, Somalia, Ethiopia)^#^	Adult	212	Retrospective register-based	diabetes, CHF, asthma, COPD, musculoskeletal disease	-
Mateen et al. 2012 [36]	Neurological disorders in Iraqi refugees in Jordan: data from the United Nations Refugee Assistance Information System	2010	Jordan	Iraq	All ages	7642	Retrospective register-based*	neurological disorders (ICD -10 diagnosis)	17
Yanni et al. 2012 [37]	The health profile and chronic diseases comorbidities of Us-bound Iraqi refugees screened by the International Organisation for Migration in Jordan: 2007–2009	2007–2009	Jordan	Iraq	All ages	18990	Retrospective register-based	hypertension, diabetes, cancer	26.8
Ipsos Market Research [38]	Second IPSOS survey on Iraqi refugees	2007	Syria	Iraq	All ages	754	Cross-sectional survey	hypertension, diabetes, asthma	17
Doocy et al. 2012 [39]	Chronic disease and disability among Iraqi populations displaced in Jordan and Syria	2008/2009	Jordan, Syria	Iraq	All ages	8,681	Cross-sectional survey	hypertension, musculoskeletal disease, CVD, diabetes	41 (In Jordan), 51.5 (In Syria)
Kim et al. 2012 [40]	The comparison of the insulin resistance and the prevalence of metabolic syndrome between North Korean refugees and South Korean	-	South Korea (Seoul)	North Korea	≥ 30 years	427	Cross-sectional survey/medical examination	metabolic syndrome	-
Mousa et al. 2010 [41]	Hyperglycaemia, hypertension and their risk factors among Palestine refugees served by UNRWA	2007	Jordan, Syria, Lebanon, OPT	Palestine	40+ years^¶^	7,762	Case series	hypertension, diabetes, dyslipidaemia	9
Mateen et al. 2012 [42]	Cancer diagnoses in Iraqi refugees	2010	Jordan	Iraq	All ages	7642	Retrospective register-based*	Cancer	2.15

Notes:

*Used UNHCR Refugee Assistance Information System (RAIS) data.

^#^Iraq 64%; Iran 22%; Palestine 6%; Afghanistan, Somalia, Ethiopia 8%.

^¶^Individuals younger than 40 years with risk factors for diabetes and/or hypertension were also screened.

CHF: Congestive Heart Failure.

COPD: Chronic Obstructive Pulmonary Disease.

CVD: Cardiovascular Disease.

ICD: International Classification of Diseases.

OPT: Occupied Palestinian Territories.

The data used for UNHCR reports were collected using the UNHCR Health Information System (HIS), which contained health information from all partners that provide health care for refugees [43]. The reports provided separate health data on urban refugees and other people of concern (PoC) living in urban settings [34]. The PoC to the UNHCR are refugees, asylum-seekers, refugees returning home, stateless people and some internally displaced populations. The figures do not include all refugees in urban places as some data were missing (Table 2).

**Table 2:** Urban refugee population, NCD prevalence and access to health care in some developing countries – Data from UNHCR annual reports

**Country (Urban area)**	**Total PoC**	Number (%) of refugees/asylum seekers in urban area*	**Origin of refugee population**	**Prevalence of NCDs in individuals older than five years**	**Access to host country primary health care**	Access to host country secondary and tertiary health care**
India (Delhi) [44]	20484	20484	Afghanistan, Myanmar, Somalia	CVD 1%, diabetes 2%, musculoskeletal disease 15%, renal disease 1%, respiratory disease 2%	yes	yes
Iran [44]	874263	852771(97.5%)	Afghanistan, Iraq	cancer 3%, CVD 10%, diabetes 8%, musculoskeletal disease 10%, renal disease 5%, respiratory disease 7%	yes	no
Malaysia (Kuala Lumpur) [44]	96691	96691	Myanmar, Sri Lanka, Somalia	cancer 2%, CVD 7%, diabetes 6%, musculoskeletal disease 13%, renal disease 3%, respiratory disease 3%	yes	no
Cameroon (Douala) [45]	65837	7000	CAR, Rwanda, DRC		yes	yes
Congo (Brazzaville) [45]	139665	7883(5.6%)	DRC, Rwanda, Angola	cancer 2%, CVD 28%, diabetes 8%, musculoskeletal disease 6%	yes	yes
DRC (Kinshasa) [45]	31281	2220(7.1%)	Angola, Rwanda, Burundi	CVD 6%, musculoskeletal disease 3%	yes	yes
Jordan (Amman) [46] (No data from 2011 fact sheet)	447332^#^ (govern. estimate)	53353	Iraq, Somalia, Afghanistan	hypercholesterolemia 45%, diabetes 16%, hypertension 19%, musculoskeletal disease (arthralgia) 11%, asthma 8%	yes	no#
Lebanon (Beirut) [46]	51927^#^	51927	Iraq, Somalia, Afghanistan	hypertension, diabetes, high cholesterol, asthma	yes	yes
Cote d'Ivoire (Abidjan) [47]	3287	3287	Liberia, Congo, DRC	-	yes	yes
Togo (Lome) [47]	No data	2345	Rwanda, DRC, Congo	CVD 3%, diabetes 1%, musculoskeletal disease 6%, respiratory disease 11%	yes	yes
Syria (Damascus) [48]	110905	110905	Iraq, Somalia, Afghanistan, Sudan	CVD 4%, diabetes 6%, musculoskeletal disease 7%, respiratory disease 2%	yes	yes
Yemen (Sanaa, Basateen) [48]	61058	45353(74.3)	Somalia, Ethiopia, Iraq	CVD 1%, diabetes 1%	yes	yes
Egypt (Cairo) [48]	44570	44570	Sudan, Somalia Iraq,	CVD 1%, diabetes 1%, renal disease 1%	yes	no
Kenya (Nairobi) [49]	548603	52472(9.6)	Somalia, Ethiopia, Congo	CVD 1%, diabetes 1%, respiratory disease 1%	yes	yes
Uganda (Kampala) [49]	108619	42500(39%)	DRC, Somalia, Eritrea	diabetes 2%	yes	yes

Notes:

*UNHCR refers to as People of Concern (PoC).

**Refugees pay the same amount for national/government secondary and tertiary health care as host country nationals.

^#^2008 data; including both registered and unregistered Iraqi refugees.

CVD: Cardiovascular Disease, DRC: Democratic Republic of Congo.

Overall, the prevalence of NCDs was high among urban refugees in the Middle East, ranging from nine per cent to 50 per cent [34,35,37-39,41], compared to the prevalence among urban refugees in Asia and Africa, where the prevalence was between one per cent to 30 per cent [34,35,40]. The most prevalent NCDs among urban refugees in developing countries were musculoskeletal disease and pain problems, CVD, diabetes and chronic respiratory disease. Cancer and renal disease were reported less frequently. Access to health care varied considerably depending on the country of asylum and not all the reviewed studies assessed urban refugees’ access to health care.

### Hypertension and other CVDs

Self-reported hypertension among Iraqi refugees in the Middle East ranged between 3.3 per cent and 30 per cent [38,39]. During screening prior to resettlement in a third country, the prevalence was found to be 33 per cent [37]. Screening of Palestinian refugees in some countries in the Middle East for high blood pressure showed that 18.7 per cent had high blood pressure (≥140/≥ 90 mmHg), with considerable comorbidity with other NCDs [41]. In Iran, where the majority of refugees are from Afghanistan and 97 per cent of them are in urban Iran, the prevalence of CVD disease was ten per cent [44]. Seven per cent of urban refugees in Malaysia had cardiovascular problems; more than 90 per cent of this population was from Myanmar. In Africa, the prevalence was one per cent in urban Kenya, three per cent in Togo and 28 per cent in Congo [45].

### Chronic pain and musculoskeletal disease

A prevalence of musculoskeletal disease ranging from three per cent to six per cent was reported in certain urban refugees in Africa (n = 12,448) [45]. This group was mainly from Democratic Republic of the Congo (DRC) and Rwanda (Table 2). Elsewhere, diagnoses based on International Classification of Diseases (ICD-10) identified back pain (5.2 per cent) and headache (2.3 per cent) as the main chronic pain complaints among Iraqis refugees in Jordan [36]. Arthralgia (11per cent) was also reported in this population [46]. In Syria, 16.6 per cent of adult Iraqi refugees suffered from musculoskeletal disease [39]. The prevalence of musculoskeletal disease in urban refugees in India, Iran and Malaysia was 15 per cent, ten per cent and 13 per cent, respectively [44]. The majority of urban refugees in these countries are from Afghanistan or Myanmar, with a few from Iraq, Somalia and Sri Lanka.

### Diabetes and metabolic disorders

Screening of diabetes mellitus in Palestinian refugees (n = 7,762) in Middle East countries (Jordan, Syrian Arab Republic, Lebanon, the West Bank and the Gaza Strip) revealed that 9.8 per cent of those older than 40 had high blood glucose levels [41]. For Iraqi refugees, the prevalence of self-reported diabetes in adults in Jordan was 9.1 per cent [39] and in Syria was 2.5 per cent [38] and 7.6 per cent [39], whereas in Jordan, screening for resettlement revealed a rate of 2.7 per cent among all age groups [37]. Refugees from DRC, Rwanda and Angola living in urban Congo had a higher prevalence of diabetes (eight per cent), compared to one to two per cent among other refugees in African urban centres [45]. In Malaysia and Iran, the prevalence was six per cent and eight per cent, respectively [44]. The prevalence of thyroid gland diseases was less than one per cent among Iraqi refugees in Jordan and Iraqi, Iranian, Palestinian, Somali and Ethiopian refugees in Ankara [35,37]. The prevalence of metabolic syndrome among North Korean refugees in Seoul (South Korea) was 20.8 per cent in men and 15.3 per cent in women [40], which was slightly higher than the prevalence among South Korean population (24.8 per cent in 17.5 per cent in women) [40].

### Cancer

The prevalence of cancer among urban refugees was less than one per cent to two per cent. Most of the studies involved Iraqi refugees in the Middle East [37,38,42]. The prevalence was 1.3 per cent [39] and 2.15 per cent [42] among Iraqi refugees in Jordan [42]. The prevalence among Iraqi refugees screened for resettlement was also 1.3 per cent [37]. A cancer rate of less than one per cent (0.34 per cent) was reported in a purposive sample of Iraqi refugee households (n = 3,553) in Syria [38]. Two per cent of refugees in Malaysia had cancer [44]. In Africa, two per cent of urban refugees in Congo (Brazzaville) had cancer [45]. There were no reports of cancer in other countries in Africa and Asia.

### Chronic respiratory disease

Various rates of chronic respiratory disease, including asthma and chronic obstructive pulmonary disease (COPD), were reported among refugees in Asia (including the Middle East) and Africa. The prevalence of chronic respiratory disease was 3.1 per cent and six per cent in adult Iraqi refugees in Jordan and Syria, respectively [39]. Among refugees in Jordan, the prevalence of asthma was 1.4 per cent [38]. In Turkey (Ankara), the prevalence of asthma was three per cent and the prevalence of COPD was two per cent among Iraqi, Iranian, Palestinian, Afghan, Somali, and Ethiopian refugees [35]. The prevalence of chronic respiratory disease among refugees over five years of age in Africa was between two per cent to 11 per cent [44-49]. A prevalence of seven per cent was reported in Iran [44].

### Other NCDs and related risk factors

Other NCDs include kidney disease, neurological problems and cerebrovascular diseases. Mateen et al. [36] reported a prevalence of 12.5 per cent of chronic neurological diseases among Iraqi refugees (n = 7,642) in Jordan, of which 1.3 per cent were cerebrovascular diseases including stroke. The prevalence of epilepsy was 2.2 per cent in Mateen’s study. However, the prevalence of epilepsy was only 0.2 per cent in Iraqis screened for resettlement [37]. The prevalence of chronic kidney disease among Iraqi refugees living in Jordan and Syria ranged from 0.5 per cent to three per cent [38,39], when screened for resettlement the prevalence was 0.04 per cent (n = 18,990) [37]. Five per cent and three per cent of refugees older than five years in Iran and Malaysia, respectively, were diagnosed with kidney disease [44]. Of the Iraqi refugees in Jordan, 4,495 (38 per cent) were overweight (Body mass index (BMI) = 25.5–29.9 kg/m^2^) and 3,982 (34 per cent) were obese (BMI > 30 kg/m2) [37]. Among the same group, 12.4 per cent were current smokers [37]. Many Iraqi refugees had at least two chronic conditions or risk factors, including hypertension, diabetes and obesity [37]. Obesity among Palestinian refugees in Syria, Lebanon, Jordan and the occupied Palestinian territories (OPT) ranged from 22.4 to 53.7 per cent [41]. More than 40 per cent of Palestinian refugee men were smokers [41]. Among North Korean refugees in Seoul (South Korea), abdominal obesity in men aged between 30 and 39 was 5.6 per cent [40].

### Access to health care

Access to health care is defined as a timely access to medical services for NCD that are affordable, acceptable and meet the needs of most urban refugees, whether provided by the host governments, UNHCR and its partners, other aid organisations or private providers. Refugees sought primary, secondary and tertiary health care for NCD. Private, public, UNHCR and nongovernmental organisation (NGO) clinics provided health care to urban refugees. Palestinian refugees in the Middle East had access to health care services at all levels [41]. Nearly 60 per cent of Iraqi refugee households in Jordan and Syria were able to access primary health care, of which more than 40 per cent were able to see medical specialists [39]. Iraqi refugees mainly received their health care from the private sector in Jordan and from the Syrian Red Cross in Syria [39]. Medications for all diseases, particularly chronic illnesses, were not affordable to many Iraqi refugees [36,38]. For the majority of refugees, cost was the main barrier to accessing health care [38,39,44,48]. In countries where public health was offered to refugees, access to health care was reported to be acceptable. In many developing countries, urban refugees could access primary, secondary and tertiary health care services in the same way as nationals [45-49]. In Egypt, Malaysia and Iran, refugees were either charged the full foreigner fee or a reduced fee in order to access secondary and tertiary care in the public sector. In all cases, the rate was higher than that for the local people [44]. Referrals from primary care to the upper level of health care, although limited, were available for refugees using UNHCR and its partner services [35,36,44-49]. Diagnostic testing (e.g., magnetic resonance imaging, electromyogram, electroencephalogram and computed tomography scan) was also available to Iraqi refugees in Jordan [36]. In most countries, UNHCR has medical referral committees to assist refugees in obtaining specialised care. UNHCR has also initiated health insurance schemes that cover chronic diseases in Iran and West Africa [44,47].

## Discussion

### Methodological issues in refugee research

Different methods were used to measure NCD prevalence in the reviewed articles. Similar to other studies of urban refugees, these studies feature methodological shortcomings, including the difficulty of accessing refugees and obtaining a representative sample, data collection constraints, the accuracy of data and the use of self-report questionnaires, which are biased by overestimation or underestimation of disease symptoms. For instance, one study found that Iraqi refugees were more likely to self-report diabetes than other refugees [50]. Sampling bias, different data collection methods and tools, cultural and language barriers and ethical considerations are concerns in refugee research that usually affect the findings and these studies are no exception [51,52]. There was also variability in definitions of NCDs among the studies and only two studies used the ICD classification of diseases [36,42]; the other studies either developed their own definitions of NCDs cases or did not mention any.

Some studies included refugees in all age groups, whereas other studies included only adult refugees. However, because most of the studied refugees were Iraqis, the findings cannot be generalised to all refugee populations, especially when considering the limitations mentioned above. Moreover, the studies in Turkey and North Korea included small groups of urban refugees. Nonetheless, the study by Doocy et al. [39] represents a good effort to overcome the challenges of refugee research in urban settings. Although the UNHCR used its standardised HIS forms that broadly categorise chronic diseases into reporting categories (with reference to ICD 10), the data were collected by different partners, which may affect its quality. The data, however, remain the most updated information on refugee demographics.

Unfortunately, there is no generally agreed upon method that is best suited for refugee studies, although some tools that have been used in the general population have been adapted to refugee research, such as the Harvard Trauma Questionnaire and the Hopkins Symptom Checklist. It is anticipated that community-based studies that include prospective examinations of refugee health issues would be more practical in refugee populations residing outside of camps [2]. Given the heterogeneity of refugees in most urban settings, organising them into communities (e.g., by country of origin) would improve communication with and access to refugee populations.

### Prevalence of NCDs among urban refugees

Noncommunicable diseases have become the primary burden of disease that affects the populations of many countries, but developing countries are affected the most owing to both socio-economic transitions and changes in the burden of disease [11,13]. Similarly, there is a growing recognition that NCDs represent a new challenge in refugee operations [2,3,9]. Three of the priority NCDs (as defined by the WHO): CVD, diabetes and chronic respiratory disease, have been reported in urban refugee populations. Cancer has not been reported for most refugees, possibly because the diagnosis of the disease requires a thorough examination, that might not be available for many refugees.

#### The Middle East

The burden of chronic disease conditions among Iraqi refugee has been highlighted in the operations for this heavy refugee caseload [19] and has drawn the attention of the international community to new challenges in health service delivery to urban refugee. The percentage of elderly Iraqi refugees is consistent with that of middle-income populations. Ageing is a driving force for NCDs and disproportionately affects older people, although mortality in developing countries is higher among those under 60 years of age [11]. Hypertension affects one-fifth to one-third of Iraqi refugees, a rate higher than that in the Iraqi population in Iraq (prevalence 4.2 per cent) [53]. Other evidence has shown that Iraqi refugees suffer from diabetes and asthma more than many other refugees [50]. Displacement may result in increased morbidity or the worsening of existing conditions [54,55]. The variation in the prevalence of some NCDs among Iraqi refugees may be explained by the difference in study designs and refugees’ expectations of the outcome of the study (e.g., resettlement in a third country). The Middle East is known to have high rates of major NCDs, and NCDs and their risk factors are the main causes of morbidity and mortality [56]. The increased NCD prevalence is attributed to the social, economic and lifestyle changes that are occurring in the region. This may be true for Palestinian refugees who become part of the urban life in the Middle East and whose health profiles feature more NCDs [41]. As the international community struggles to meet the education, shelter and food needs of Iraqi refugees, chronic diseases will remain a major health issue.

#### Africa

No studies on NCD prevalence amongst urban refugee populations in Africa were found, except for the routine data collected and analysed by UNHCR. This may be because communicable diseases are more prevalent among refugees in Africa [19] and require more attention than NCDs. Refugees in Africa are of mixed nationalities and NCD prevalence information does not refer to specific groups or nationalities. The considerable prevalence of CVD, respiratory disease and diabetes among urban refugees in Congo, Togo and DRC is possibly due to the ongoing transitions in disease profiles towards NCDs, the lifestyle changes, urbanisation and the ageing of populations [12]. To understand the consequences of the changes that are occurring, an assessment that takes into account country of origin, country of asylum and refugee demographics is important. However, demographic information is not available for many refugees in Africa [4]. In UNHCR reports, the prevalence of NCDs is for refugees who received health care and may include other PoC, which means the results should be interpreted cautiously.

#### Asia

Asia hosts the largest refugee population in the world. Many refugees live in urban Iran, Pakistan, India and Malaysia [1]. In Asia, a few studies have found that pain disorders and chronic gastrointestinal diseases are prevalent among urban refugees [30,57]. Symptoms of non-specific pain and backaches among refugees may arise in those who have experienced trauma or torture [58]. Refugees are also most likely to engage in hard labour work to generate some income. The evidence also shows that urban refugees in Asia are more likely to suffer from cardiovascular and renal disease than refugees in Africa. Burmese refugees in urban Malaysia have a prevalence of hypertension of 14.8 per cent [50], which is less than their home country’s average of 29 per cent [59]. This is because many Burmese refugees are young adults in the age range of 20-45 years, with a smaller population older than 45 years. In Pakistan, Bangladesh, Thailand (and possibly other countries in Asia), there may be urban refugees who remain unaccounted for. For example, many Afghan children do not have access to urban diagnosis centres in Pakistan; however, those who do have access have been found to have various types of cancer [60]. In South Korea, the upcoming result of a study of North Korean refugees’ health status is expected to answer questions about NCD incidence in this group [61].

Resettlement countries perform medical examinations on refugees within the first few months of arrival to determine their health status. The focus of the medical screening in many resettlement countries is on infectious diseases. And although data on refugees admitted for resettlement in a third country may not be separated by the type of refugee setting (e.g., camp, urban), knowing the country of first asylum or refugees’ nationalities can help to determine the type of refugee setting before arrival. Among Iraqi refugees who resettled in the United States, the prevalence of diabetes and CVD were similar to the findings presented here, as were their risk factors, such as smoking and obesity [50,62]. NCDs were also common among refugees resettled from Africa [63]. For instance, Somali refugees who had been living in Kenya and were recently resettled in the United States were found to have high rates of hypertension (up to 30.5 per cent) in the 45 and older age group [32,50]. A large group of Somali refugees are in camps in Kenya, but considerable numbers live in urban Kenya and are reported to have age-related illnesses [64]. Other evidence suggests that the prevalence of NCDs such as hypertension and diabetes is higher in refugees who experienced more traumatic events than in low-trauma refugees [65] and is affected by refugees’ region of origin [66]. Thus, refugees who come from countries or regions with a known burden of NCD require more than infectious disease screening and immunisation record checks. Healthy start programmes in resettlement countries should actively encourage refugees to seek medical advice and keep medical appointments for chronic conditions. After resettlement, refugees must address other concerns that may make health issues a low priority [63,67].

### Comparison of NCD prevalence among urban refugees with the prevalence in their home countries

To better know the diseases that are expected in a specific refugee group, it might be useful to examine the country of origin’s disease profile and the social and economic changes that are occurring. Experiences with conflicts in middle-income countries have shown what diseases to expect among refugees fleeing from these countries. Table 3 presents data on income level and ageing in some refugee home countries in relation to the prevalence of select NCDs. For the majority of these countries, data on NCDs and health systems responses to the growing problem of NCDs are limited [68,69]. The information in Table 3 also shows that in some refugee home countries (e.g., Sudan, Somalia, Congo and Myanmar), the prevalence of diseases such as hypertension is higher than the regional average [70]. Some of these countries (Angola, Cote d’Ivoire, Congo and Sudan) have become middle-income nations and are reported to have increased prevalence of NCDs and ageing populations [71,72]. Angola, Rwanda, Congo and Cote d’Ivoire have larger populations aged 60 and above than the regional averages [71]. In the case of Iraqi refugees, the information in Table 3 supports other evidence on NCD prevalence [2,50].

**Table 3:** NCD prevalence, percentage of ageing population and income level of some refugee home countries

**Country**	**% raised blood pressure (aged 25+) (2008) (reg. average)**[70]		**% raised blood glucose (aged 25+) (2008) (reg. average)**[70]		**% obesity (aged 20+) (reg. average)**[70]		**% of population aged ≥ 60 years, 2009 (reg. average)**[71]	**Income level**[72]
	**Male**	**Female**	**Male**	**Female**	**Male**	**Female**		
Afghanistan	27.2(30.7)	27.9(29.1)	8.9(11.0)	9.5(11.6)	1.5(13.0)	3.3(24.5)	3.8(7.1)	low income
Iraq	30.1(30.7)	28.7(29.1)	12.7*(11.0)	12.5*(11.6)	22.3*(13.0)	36.2*(24.5)	4.7(6.9)	lower-middle income
Somalia	39.9*(30.7)	35.7*(29.1)	7.9(11.0)	7.7(11.6)	3.4(13.0)	7.1(24.5)	4.3(4.7)	low income
Sudan	39.9*(30.7)	33.5*(29.1)	8.6(11.0)	8.1(11.6)	24.0(13.0)*	2.0(24.5)	5.7(7.0)	lower-middle income
DRC	38.5(38.1)	33.33(35.5)	6.6(8.3)	7.8(9.2)	0.7(5.3)	3.0(11.1)	4.2(4.5)	low income
Rwanda	43.6*(38.1)	40.2*(35.5)	6.7(8.3)	6.1(9.2)	4.9(5.3)	4.0(11.1)	18.6*(4.5)	low income
Angola	39.6*(38.1)	33.8(35.5)	8.2(8.3)	8.7(9.2)	3.8(5.3)	10.2(11.1)	17.2*(7.0)	upper-middle income
Ethiopia	33.0(38.1)	28.3(35.5)	7.3(8.3)	7.0(9.2)	0.9(5.3)	1.6(11.1)	5.0*(4.7)	low income
Congo	40.3*(38.1)	36.1*(35.5)	7.8(8.3)	8.5(9.2)	2.8(5.3)	7.5(11.1)	19.3*(4.5)	lower-middle income
Cote d’Ivoire	41.6*(38.1)	35.7*(35.5)	9.2*(8.3)	9.7*(9.2)	3.9(5.3)	9.7(11.1)	19.3*(4.8)	lower-middle income
Myanmar	34.4*(25.4)	29.2*(24.2)	6.1(9.9)	7.1(9.8)	2.0(1.7)	6.1(3.7)	8.0 (8.5)	low income

Note: Diabetes prevalence in this table was based on a fasting glucose blood sample and not self-reported prevalence.

Reg. average: Regional average.

*Values are greater than the regional average.

DRC: Democratic Republic of the Congo.

Age-related and other types of chronic diseases in urban Africa are expected to rise [12,73], as countries face epidemics of both communicable and noncommunicable diseases. The ageing of people and the change in socio-economic status in refugee-producing nations are among the reasons for the projected rise in NCDs among refugee populations. Other than refugees in the Middle East, urban refugees in the DRC (refugees are from Angola, Rwanda, Burundi) and Angola have the highest percentage of elderly compared to other urban refugees in Africa [71,74]. This fact may explain the increased morbidity from NCDs in this group of refugees while highlighting the need for greater attention to age-related diseases among refugees. There was no difference between the percentage of Iraqi refugees who are over 60 years of age and the percentage in Iraq [71]. The assumption is that urban refugees are mainly young men, but considerable numbers of other age groups, including the elderly, are also observed in urban areas. Refugees from middle-income countries are generally older and refugees in protracted situations are also expected to reach older ages [2]. UNHCR estimated in 2011 that refugees in protracted conditions represented almost 70 per cent of total refugee populations [1]. Of all refugees, the percentage of the elderly of two per cent to four percent [4] seems incongruous when categorising refugees by country of origin; at the same time, it emphasises the need to look beyond average data.

### Risk factors for NCDs

Most NCDs have known risk factors that can be targeted by preventive and health education interventions. Risk factors such as unhealthy diet, tobacco use, sedentary lifestyle and excessive alcohol consumption contribute to the development of NCDs. Moreover, urban environments in developing countries are difficult for refugees, who usually live in poor housing conditions, which increase the risk of developing NCDs such as chronic respiratory disease. Noncommunicable diseases require opportunistic case finding, early detection, identification of high risk status and long-term follow-up [75]. The preventive aspect is not easily exploited or is overlooked in the context of urban refugees. The disorganised nature of urban refugees and limited resources further complicate this task. Other determinants, such as individual and genetic factors, environmental factors, country of origin and lifestyle, also relate to the development of NCDs. Palestinian and Iraqi refugees have higher NCD risk factors (smoking and obesity) than other refugees in developing countries [41,70]. Palestinian refugees who have been living in urban settlements in the Middle East for decades may have adopted lifestyles that contribute to NCDs similar to the populations in these countries [56]. Overweight and obesity are common among adult Iraq refugees and are comparable to obesity rates in the Iraqi population in Iraq [50,53]. Additionally, a study showed that Iraqi refugees have the highest prevalence of chronic disease comorbidities among refugee populations [50]. Smoking and other CVD risk factors are also common in refugees from South East Asia [76] and from Bosnia [77]. Although refugees are mobile populations with changing demographics, the risk of developing an NCD could be evaluated when refugee seek medical services.

### Access to health care

Urban refugees’ access to primary health care varied depending on the country of asylum, and not all the reviewed studies assessed refugees’ access to health care. Overall, urban refugees have fairly good access to primary health care provided by the public health facilities in host countries, UNHCR-supported clinics, other aid organisations and private clinics. However, access to secondary and tertiary health care is problematic. Some urban refugee populations need to be made aware of the available health care options, while others (e.g., Iraqis) left high-quality health care back home and the health services they receive may not address the demand for continuous care that chronic conditions require. It is likely that in many developing countries, refugees who access public health facilities are registered as foreigners, which means for a number of refugees, information on access to health care at all levels may be missing. Furthermore, the data on access to health services reported by most of the reviewed studies and reports are best described as health care utilisation rather than health care need. Given that some urban refugee groups were not accessible or data were missing, means that the UNHCR data represent only refugees benefited from health services provided by UNHCR and its partners. Dependence on UNHCR for health care remains the only option for the majority of urban refugees, yet not all refugees have access to this assistance [19]. The fact that a number of first asylum countries, including Jordan, Syria, Lebanon, Pakistan, Malaysia, India and many other countries in Asia, have not ratified the refugee convention has implications for refugees’ access to health care. Consequently, some of these countries make urban life unfavourable for refugees or in the best scenario, turn a blind eye to their health care needs. International organisations may not be allowed into the country to assist urban refugees (e.g., Malaysia). These recognition and protection issues negatively affect urban refugees’ access to health care. In some cases, negotiations between UNHCR and governments have resulted in improvements in access to national public health services [44]. In Africa, although urban refugees do not have the problem of legal presence, many countries have their own challenges in providing health care for their populations, and programmes and interventions to limit the growing NCD prevalence are still in the infancy stages. Countries must balance the provision of care to those suffering from NCDs with the ongoing fight against infectious diseases. This may lead to urban refugees’ inadequate access to health care.

Secondary and tertiary health care, when available to urban refugees, are usually prohibitively expensive, particularly in countries where refugees have to pay more for health care than nationals do. The cost of medical care is the main reason why refugees do not seek health care for NCDs [7,25,29,30,57]. Financial barriers also limit physical access to health care if refugees have to pay to travel to clinics away from their residences. The cost to treat NCDs in developing countries appears in the form of user fees, out-of-pocket payments and the cost of drugs, which limit access to treatment for many people [68]. In addition to financial barriers, urban refugees’ access to health care is limited by geographic accessibility, security and cultural and language barriers [78]. Even in countries where refugees have access to the host country’s public health care, the health system is usually overstretched or of low quality, as in many African countries. Although the private sector in developing countries is more accessible to the poor [68], refugees are more financially disadvantaged than the urban poor and cannot access private clinics. UNHCR and other humanitarian aid agencies working with limited financial resources find it difficult to meet the increasing cost of urban refugee health care. One exception is Palestinian refugees, who have access to NCD preventive and curative care provided by the United Nations Relief and Works Agency for Palestine Refugees in the Near East (UNRWA) [79], which has responsibility separate from UNHCR. In addition to the said accessibility problems, inability to speak the language of health care providers may result in delays in seeking care or in the delivery of an inappropriate care. However, working with refugee communities has helped UNHCR to overcome the language barriers in countries such as Malaysia. Inability to communicate with local people also subjects refugees to discrimination and xenophobia, leading them to avoid public places and to hide. In other situations, security and protection issues limit accessibility to health care [52,80] because law enforcement authorities in some countries reportedly do not distinguish between refugees and other migrants. As a result, refugees avoid travelling to clinics downtown, where they may come in contact with local authorities.

The implementation of refugee health insurance schemes by UNHCR in Iran and West Africa is expected to further improve refugee access to specialised care [81]. The refugee health insurance initiative could be emulated in other countries where refugee’s access to health care is problematic. To do so, it is important to identify effective ways to reach the most vulnerable refugee populations to participate and to find suitable ways to pay for those who cannot afford premiums. One of the approaches deemed appropriate to improve the management and control of NCDs in developing countries is the integration of the delivery of care for communicable and noncommunicable diseases at the primary care level [69,82]. Refugees are expected to benefit from this integration especially in countries where the integration of refugee health care into the local health system is in place or feasible. Then, with support for existing facilities (with human and financial resources), access to health care could improve. Without permanent solutions for millions of refugees, the demand for health care for the growing number of urban refugees is expected to increase in the coming years.

### Study limitations

There are limitations to this study. First, many of the studies and reports included in the review were based on retrospective data, some of which were collected for other services provided to refugees and not primarily for research or epidemiological purposes. Second, cross-sectional surveys collected self-reported information or self-reported and medical examination data on chronic diseases, with the potential for overestimation or underestimation of health conditions. Third, different definitions and classifications of NCDs were adopted in the studies and reports, which could have affected the prevalence rates, making comparisons between studies and regions and/or countries difficult. Two studies were based on the UNHCR Refugee Assistance Information System (RAIS) database, which uses ICD-10 as diagnostic tool; however, data accuracy was limited because diagnoses were made by physicians and other health care workers. Fourth, it was difficult to ascertain that all data from the studies or the reports were exclusively on urban refugees. For instance, the majority of Iraqi refugees in the Middle East live in urban areas, although the city or urban area is not always stated and not all refugees are registered. Unregistered Iraqis also benefit from UNHCR health care assistance. Similarly, UNHCR data report all PoC to the UNHCR in an urban setting regardless of refugee or asylum-seeker status. Fifth, our search was limited to studies in English only and it is possible that important studies in other languages were omitted. Likewise, there may be unpublished reports from other refugee organisations that were not included. Finally, it would be more appropriate to include post-arrival (resettlement) screening results to increase the pool of studies, although such data are inclusive of all refugees without noting their origin.

## Conclusion

The study found few research articles on NCD prevalence among refugees living in the urban areas of developing countries. Many of these studies were among Iraqi refugees in the Middle East. The UNHCR reports covered most urban refugees, although some refugees remain unaccounted for due to the limited resources available to attend to their chronic health needs or the loss of contact with them. Generally, the prevalence of NCDs among urban refugees varied depending on refugees’ region or country of origin. However, owing to the limited number of articles and the methodological biases of the heterogeneous literature included in the review, the observed prevalence (e.g., in the Middle East) may not reflect the actual trend in NCD prevalence among urban refugees in developing countries. Hypertension, musculoskeletal disease, diabetes and respiratory disease were the major diseases observed. Urban refugees in developing countries have adequate access to primary health care services, but access to secondary and tertiary health care remains problematic for some refugees. Financial barriers are the number one reason why urban refugees do not seek health care. The fact that many refugee hosting countries face difficulties in delivering health care to their own populations and lack strategies to appropriately address NCDs means refugees’ access to health care is also unlikely. Nonetheless, the UNHCR and its partners, governments and other refugee organisations should use primary health care wherever available as an opportunity to detect NCDs among urban refugees early and to provide appropriate care. With the recent conflicts in middle-income countries (e.g., Syria and Libya), the health profile of refugees is expected to be similar to that observed in Middle East refugees and most are expected flee to cities. Options and priorities must be identified to improve urban refugees’ access to available health care resources, to finance health care for refugees and to advocate for such financing. Researchers must overcome methodological and logistical problems, security issues and cultural and language barriers and must minimise sampling biases before they can produce sound research results and more solid conclusions. It is hoped that these findings will raise awareness of the need for consolidated efforts to provide health care for urban refugees and will stimulate the conduct of more research to highlight the burden of NCDs among urban refugee populations in developing countries.

## Abbreviations

BMI: Body mass index; CHF: Congestive heart failure; COPD: Chronic obstructive pulmonary disease; CVD: Cardiovascular disease; HIS: Health Information System; HIV: Human Immunodeficiency Virus; ICD: International classification of diseases; IOM: International Organisation for Migration; NGO: Non-governmental Organisation; NCD: Noncommunicable disease; OPT: Occupied Palestinian Territories; PoC: People of concern; RAIS: Refugee assistance information System; UN-HABITAT: United Nations Human Settlements Programme; WASH: Water, sanitation and hygiene.

## Competing interest

We declare that we do not have competing interests.

## Authors’ contributions

Both authors conceived the idea. AHA conducted the search and SMJ reviewed the articles and reports against the inclusion criteria. AHA drafted the manuscript and SMJ revised and edited the manuscript. All authors read and approved the final manuscript.

## Authors’ information

AHA is a student in the Master’s of International Health at the Institute of Tropical Medicine and International Health – Charité Medical University Berlin. SMA is a Professor of Health Economics and Consultant in Public Health Medicine. He is the Senior Research Fellow at United Nations University International Institute for Global Health. He is also the head of International Centre for Casemix and Clinical Coding of Universiti Kebangsaan Malaysia. SMA is a co-chair of Morbidity Technical Advisory Group of ICD-11 Revision of World Health Organisation-Family of International Classification.
